# Screen and Verification for Transgene Integration Sites in Pigs

**DOI:** 10.1038/s41598-018-24481-1

**Published:** 2018-05-09

**Authors:** Linyuan Ma, Yuzhe Wang, Haitao Wang, Yiqing Hu, Jingyao Chen, Tan Tan, Man Hu, Xiaojuan Liu, Ran Zhang, Yiming Xing, Yiqiang Zhao, Xiaoxiang Hu, Ning Li

**Affiliations:** 1 The State Key Laboratory for Agricultural Biotechnology, College of Biological Science, China Agricultural University, Beijing, 100193 China; 2 Beijing Advanced Innovation Center for Food Nutrition and Human Health, China Agricultural University, Beijing, China

## Abstract

Efficient transgene expression in recipient cells constitutes the primary step in gene therapy. However, random integration in host genome comprises too many uncertainties. Our study presents a strategy combining bioinformatics and functional verification to find transgene integration sites in pig genome. Using an *in silico* approach, we screen out two candidate sites, namely, Pifs302 and Pifs501, located in actively transcribed intergenic regions with low nucleosome formation potential and without potential non-coding RNAs. After CRISPR/Cas9-mediated site-specific integration on Pifs501, we detected high EGFP expression in different pig cell types and ubiquitous EGFP expression in diverse tissues of transgenic pigs without adversely affecting 600 kb neighboring gene expression. Promoters integrated on Pifs501 exhibit hypomethylated modification, which suggest a permissive epigenetic status of this locus. We establish a versatile master cell line on Pifs501, which allows us to achieve site-specific exchange of EGFP to *Follistatin* with Cre/*lox*P system conveniently. Through *in vitro* and *in vivo* functional assays, we demonstrate the effectiveness of this screening method, and take Pifs501 as a potential site for transgene insertion in pigs. We anticipate that Pifs501 will have useful applications in pig genome engineering, though the identification of genomic safe harbor should over long-term various functional studies.

## Introduction

New genes are routinely introduced to mammalian cells to elucidate their expression pattern and function. However, this is often achieved by random integration into the genome, either by viral transduction^1,2^ or plasmid transfection. Viral vector insertion sites exhibit a strong bias towards actively transcribed genes and can disturb endogenous gene expression^3^. Uncontrolled transgene integration can lead to several undesirable effects, including unpredictable expression and unexpected interactions between integrated exogenous DNA and the neighboring chromatin environment^4,5^. It would be also problematic once transgenes activate oncogene expression^6^. Moreover, exogenous DNA connecting into a large tandem repeat structure is readily subject to repeat-induced gene silencing^7^.

Due to anatomic^8^, metabolic^9^, neurobiological^10^, and physiological^4^ analogy between pigs and humans, pigs are extensively utilized in a wide range of biomedical researches as a model animal. Precise and safe genetic modification in pigs facilitates the establishment of a human genetic disease model, promotes the study of gene expression dynamics to elucidate molecular mechanisms, and also benefits the agricultural application. Researchers have invested much effort to overcome the disadvantage of random DNA integration induced variable transgene expression and insertional oncogenesis. A lot of work is focused on the design of vectors with better transgene expression^11^.

As an alternative approach, precise integration of transgenes to specific safe locations in pig genome would help to solve the problem of random integration. At present, the most efficient methods available for targeted gene delivery are based on homologous recombination (HR)^12^. Double strand breaks (DSB) have been shown to stimulate homologous recombination by more than 10,000-fold in cultured cells^13^. In addition, we can achieve highly efficient site-specific integration by DSB mediated homologous recombination through taking advantage of recently emerging pioneering technologies, including zinc finger nucleases (ZFNs)^14^, transcription activator-like effector nucleases (TALENs)^15,16^, and the clustered regularly interspaced short palindromic repeats (CRISPR)/CRISPR associated (Cas) system^17^.

Nevertheless, determining precisely where to integrate exogenous DNA sequences in pig genome to maximize transgene safety has received little attention. Genomic safe harbor (GSH) can support stable and reliable transgene expression in at least several different cell types without detectable adverse consequences^18^. However, there is little research on the screen of genomic locations and their suitability of transgene knock-in. Strategies for screening safe harbors have also been suggested. For instance, transgenes are integrated into the genome randomly first, and then integration sites are chosen that offer higher expression^19^. However, such reverse screening method needs to analyze a large amount of integration sites to obtain a suitable site. Using a comparative genomics approach, sites applied in one species could be aligned to other species based on homologous sequences between species, such as mouse *Rosa26* locus, an ideal locus widely used in mouse genetic modification. *Rosa26* locus was identified in humans^20^, pigs^21^, rabbits^22^, sheep^23^ and probably other mammals based on the conserved sequences between them. Even though *pRosa26* can support excellent transgene expression in pigs^21,24^, integration in the *pRosa26* locus indeed disrupts the gene *Rosa26*^25^. Human gene expression profiles reveal clustering of highly and constantly expressed genes to specific chromosomal regions^26^. Genes with broad tissue expression may be attractive as potential universal GSHs^18^. So, intergenic regions within epigenetic open chromosome regions might support high and reliable expression of transgene. This would be similar to the identification of intergenic H11 locus, where neighboring genes displayed broad spatial and temporal EST expression patterns^27,28^.

In this study, we propose an effective method for searching transgene integration sites combining bioinformatics and functional verification. Using gene expression data of porcine genome, we found high expression regions in the genome, and then the gene spacer regions in these regions were selected as candidate integration sites. After verification of whether the candidate loci were suitable for sustainable foreign gene expression on cellular and individual level, we considered a candidate site Pifs501 as a potential effective transgene integration site which could be used in pig genome engineering.

## Results

### Screening of high expression regions in pig genome

To find candidate genomic loci, we firstly searched the pig genome for regions where genes are highly transcribed. To achieve this, gene expression data were retrieved from a public pig gene expression altas set, including 62 tissues/cell lines^29^. Expression values from the probe sets corresponding to the same gene were averaged, and we thus obtained 62 expression values for each of the 12,997 genes after processing. Regarding expression level, a candidate region should meet two criteria: 1) the mean expression per tissue per gene within the region is top-ranked in all regions investigated; and 2) the mean of variation of expression level across tissues for all genes within the region is as low as possible. Following these criteria, a sliding-window approach was chosen. To identify a proper window size, we first checked the gene coordinates from ensemble pig genome annotations. The median transcript size of pig genes is 9.6 kb, while the size of median intergenic sequences is 23.6 kb. Consequently, we used a sliding window approach with the window size of 500 kb and a step size of 100 kb to ensure that enough genes were covered in each window. Any genes of which the transcription starting site (TSS) to transcription termination site (TTS) region overlapped with the boundary of a 500 kb window would be counted for this window. It was reported that insertional activation of neighboring proto-oncogenes led to clonal dominance or malignant transformation^30,31^. Considering cancer-related genes which might promote malignant cell transformation in the case of expression disturbance, we excluded windows containing pig homologs genes implicated in cancer in mice or humans. For each remaining window, the mean expression level across tissues for each gene was computed, and the expression values were further averaged for all genes signed for this window. Average gene expression profiles of 500 kb windows for pig chromosomes are shown in Fig. S1. Upon finishing all windows, we first selected outlier windows with high expression levels using a threshold of Q3+1.5IQR, resulting in 285 regions where genes are highly transcribed. Windows contained at least three genes as candidates. Table 1 shows the top five windows with the highest expression level, but less variation across tissues.

**Table 1:** The top five 500 kb units with high expression value.

Chr.	Start	End	Ave.	CV
chr_18	33500000	34000000	11.17282	0.411700632
chr_16	44000000	44500000	7.658157	0.32754617
chr_15	138000000	138500000	6.565982	0.355223954
chr_9	81000000	81500000	5.653846	0.909857251
chr_3	62500000	63000000	3.122311	0.365433254

### Selection of candidate intergenic regions for transgene integration

In order to avoid disturbance to transcriptional units, ideal transgene integration sites should be located at intergenic regions where surrounding genes were highly expressed. This could lead to high expression of inserted gene, but cause less adverse effects. We selected intergenic regions of the top three high expression windows (the windows were extended to 100 kb upstream since some of the window boundary are located on genes) as final candidate regions, considering both expression level and expression variations. As an incomplete annotation of eukaryotic genome, a currently considered intergenic region may contain transcripts as functional noncoding RNAs, including microRNA, tRNA, rRNA, snoRNA, etc^31^. In order to rule out this possibility, we blasted the sequences of candidate intergenic regions against Rfam-11.0^32^, rnammer-1.2^33^, snoRNA-LBME-db^34^, miRBase19^35^, and GtRNAdb^36^ database to filter out regions harboring potential functional noncoding RNAs. Intragenic regions passing the filtering criteria are listed in Table S1. All intergenic regions are listed in Table S2.

**Figure 1:**
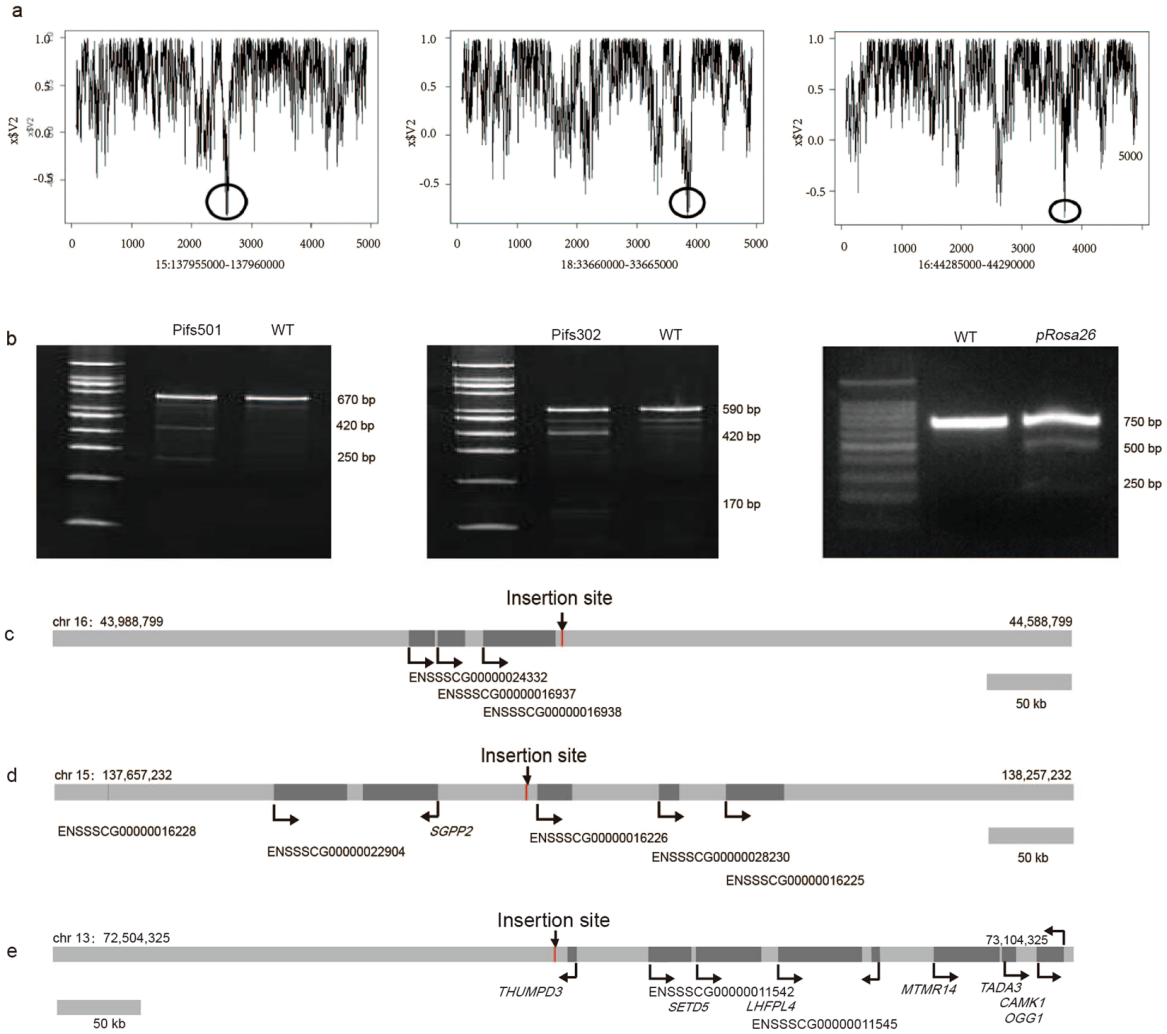
Identification of the candidate integration sites. (**a**) Nucleosome formation potential of selected intergenic regions. Areas with the smallest nucleosome forming ability are indicated by black circles. The Y-axis represents the nucleosome formation potential, while the X-axis indicates the 5 kb sequences showed at the bottom of the figure. (**b**) CRISPR/Cas9 targeting sites with higher efficiency. Detected by T7 Endonuclease I for DNA fragments containing the targeting sites. (**c**) Genomic region spanning 600 kb on Pifs501. Genes and their transcripts are shown. (**d**) Genomic region spanning 600 kb on Pifs302. Genes and their transcripts are shown. (**e**) Genomic region spanning 600 kb on *pRosa26*. Genes and their transcripts are shown.

The nucleosome formation potential of a particular DNA sequence could reflect transcription status. Actively transcribed regions are either free of nucleosomes or under dynamic nucleosome modifications or displacements^37,38^. Thus, active DNA elements are associated with open chromatin in higher eukaryotic genomes that have low nucleosome formation potential. We selected intergenic sequences without noncoding RNA potential from the three candidate regions, respectively, and calculated the nucleosome formation potential for candidate intergenic regions using the RECON web service (http://wwwmgs.bionet.nsc.ru/mgs/programs/recon/) with default settings^39^. 1 kb regions with the lowest nucleosome formation potential were preferred and indicated by black circles (Fig. 1a).

For the three intergenic regions, we planned to perform site-specific integration by applying CRISPR/Cas9 mediated homologous recombination. In order to determine the exact insertion positions, we designed a set of sgRNAs targeting multiple sites, listed in Table S3, within the candidate 1 kb regions. After detecting the targeting efficiency of all sgRNAs by T7E I, we chose the target sites with higher CRISPR/Cas9 targeting efficiency (Fig. 1b) as the final integration sites, namely, Pifs501, which lies downstream of gene ENSSSCG00000016938 on chromosome 16 and Pifs302, which lies upstream of gene ENSSSCG00000016226 on chromosome 15. The chromosomal environment of candidate integration loci, such as genes and their transcripts, are shown in Fig. 1c and d. After all of the steps described above, we obtained final candidate transgene integration sites that might be suitable to accommodate the expression of integrated DNA. Since it was reported that *pRosa26* could support ubiquitous exogenous gene expression, we selected it as a positive control, and its chromosomal environment is shown in Fig. 1e. In addition, we selected an intergenic region within a 500 kb window with lower expression level randomly, which was located downstream of TSC22D2 on chromosome 13 (named site 13), as a negative control.

### Efficient CRISPR/Cas9 mediated site-specific transgene integration

**Figure 2:**
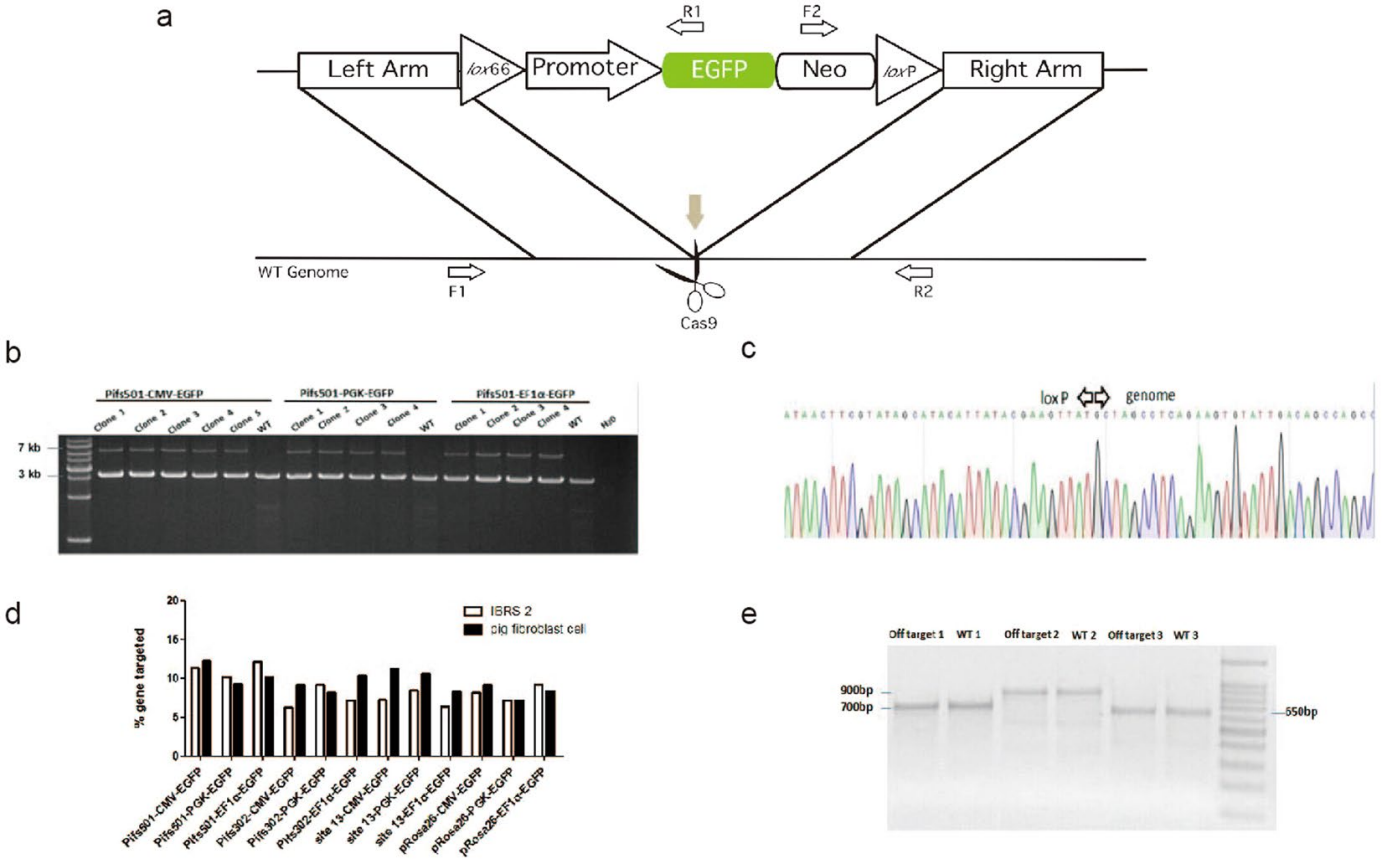
Schematics of Cas9-mediated targeted site-specific integration into candidate loci. (**a**) The targeting vectors containing EGFP expression cassette flanked by homology sequences to Pifs501, Pifs302, and *pRosa26*. Right triangles, wild-type *lox*P; left blue triangle, *lox*66. Cas9 recognition site is indicated as in A. EGFP driven by CMV, PGK, and EF1α promoter, respectively. (**b**) PCR analysis confirmed targeted integration of the indicated cassette at the Pifs501 locus. WT pig genomic DNA and water were used as negative controls. Lane 1 to lane 5 showed targeted integration of CMV promoter cassette driven EGFP cassettes; lane 7 to lane 10 showed targeted integration of PGK promoter cassette driven EGFP cassettes; lane 12 to lane 15 showed targeted integration of EF1α promoter cassette driven EGFP cassettes; lane 6, lane 11, and lane 16 were negative controls of WT genome resulting in a 3 kb band; lane 17 indicated water as a negative control. Primers spanning the whole targeting region are indicated in A (F1 and R2), resulting a ~7 kb band. (**c**) Sequencing confirmation for the 3′ junction of targeted integration of CMV-EGFP cassettes to Pifs501, sequences on the left arrow were *lox* P, and sequences on the right arrow were genomic sequence. (**d**) Targeting efficiency on different candidate sites in two cell types, IBRS-2 cell lines and pig fibroblast cells. (**e**) Off-target detection of sgRNA in the CRISPR/Cas9 system on Pifs501.

We performed functional verifications on the cellular level to determine the potential suitability for transgene expression on these two genomic locations. Since EGFP is an independent exogenous reporter gene and does not participate in the critical signaling pathway of cellular biological processes in pigs, we constructed enhanced GFP (EGFP) reporter expression vectors driven by different promoters, either from viral origin (CMV promoter) or from cellular origin (PGK promoter and EF1α promoter). Each cassette was flanked by 1~2 kb homologous sequences to the candidate sites to realize homologous recombination. The *lox* P and mutant *lox* 66 sites were arranged to flank the EGFP cassette, as indicated in Fig. 2a, which allows for the replacement of any gene of interest into the docking site by recombinase-mediated cassette exchange (RMCE) technology. We delivered the *Sal* I (NEB) linearized EGFP expression cassette and the corresponding CRISPR/Cas9 targeting vector (a total of 4 µg) to IBRS-2 which is a pig kidney cell line and pig fibroblast cells, respectively. The detection of targeted integration was achieved by PCR amplification using primer spanning the whole targeting DNA repair matrix, located on the genomic sequence outside of the homologies. Taking EGFP cassettes integrated on Pifs501 (501-CMV-EGFP) as an example, positive targeted clones are shown in Fig. 2b. The 3′ ends of the integration events were sequenced, and all were confirmed (Fig. 2c). The integration efficiency was between 6% and 15% (Fig. 2d). Single cell clone numbers were showed in Table S6. We performed off-target detection on potential Pifs501 off-target cleavage sites in the whole genome using blast and T7E I verification (Table S4). It was found that no off-target cleaving occurred (Fig. 2e).

**Figure 3:**
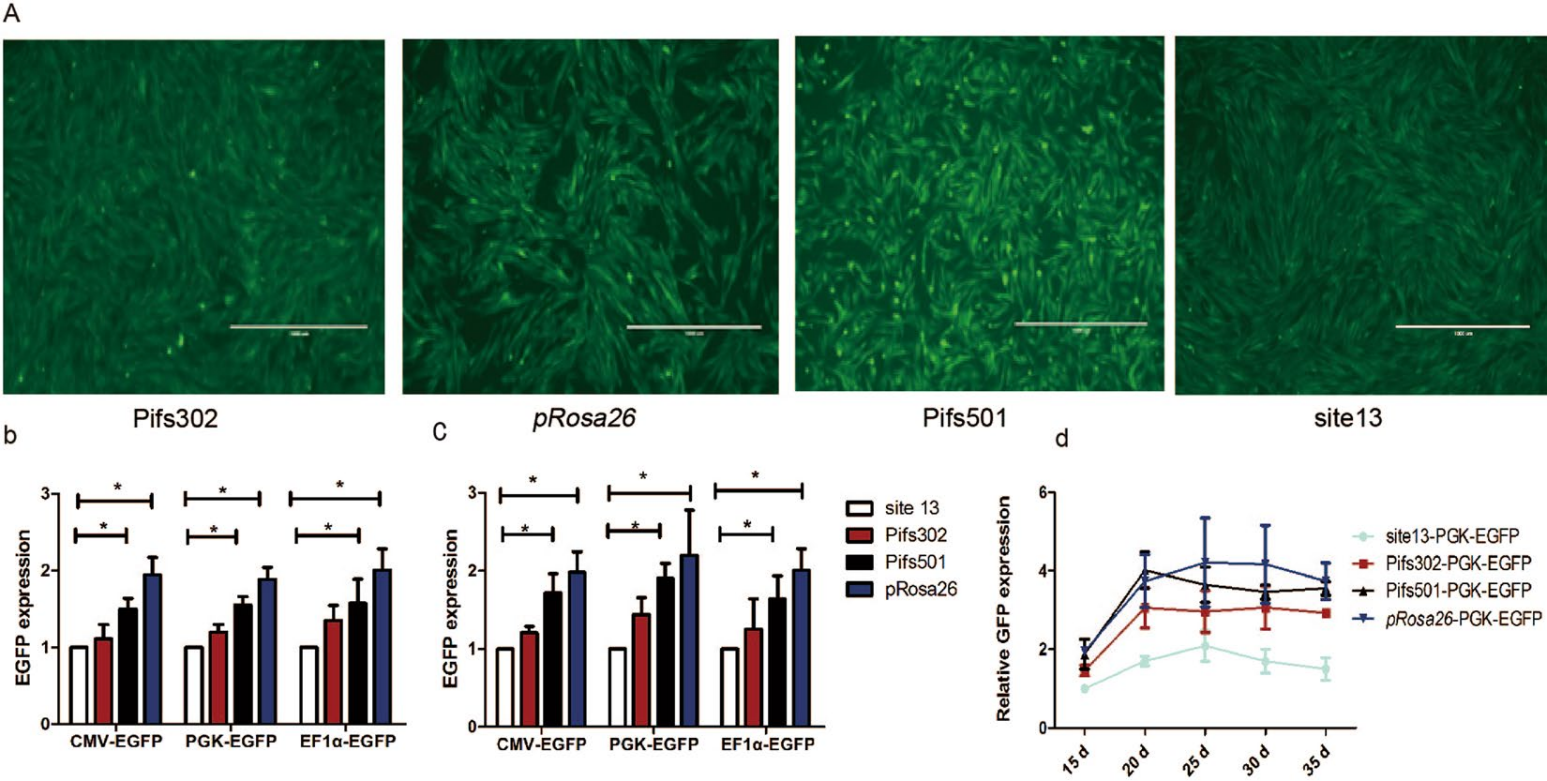
Characterization of EGFP expressions on the candidate loci. (**a**) CMV derived EGFP expression on Pifs302, *pRosa26*, Pifs501 and site 13 correctly targeted single cell–derived clones 20 d after transduction. The scale bar is 1000 μm. (**b**) EGFP expression derived by CMV, PGK, and EF1α promoters in site-specific integrated IBRS-2 cell clones on Pifs501, Pifs302, *pRosa26* and site13. Representation to the EGFP expression on site 13 by Q-PCR analysis 30 d after transduction in IBRS-2 cells (Mean ± SD, n = 3, *p < 0.05). (**c**) EGFP expression derived by CMV, PGK and EF1α promoters in site-specific integrated pig fibroblast cell clones on Pifs501, Pifs302, *pRosa26* and site 13. Representation to the EGFP expression on site 13 by Q-PCR analysis 30 d after transduction in IBRS-2 cells (Mean ± SD, n = 3, *p < 0.05). (**d**) Candidate sites support stable EGFP expression during 35 d after transduction. Representation to the GFP expression on site 13 on day 15. (Mean ± SD, n = 3)

In our experiments, full EGFP expression was observed at all of the tested loci (Pifs501, Pifs302, site 13 and *pRosa26*) with three different promoters (CMV, PGK, and EF1α promoter) in IBRS-2 cells (Fig. 3b) and pig fibroblast cells (Fig. 3c). EGFP expression with CMV promoter on four sites in pig fibroblast cells is displayed in Fig. 3a. Among the three promoters studied, EF1α promoter expression cassettes consistently displayed relative slightly higher expression at all four genomic sites, as shown in Fig. 3b and c. More importantly, the effect of integration sites on transgene expression was critical, with Pifs501 and *pRosa26* site outperforming Pifs302 and site 13 (Figs. 3b,c). It is quite likely that the chromosomal environment of Pifs501 and *pRosa26* might allow better access to transcription components. Among all the tested sites, *pRosa26* site supported the highest EGFP expression. All EGFP expressions were detected 30 d after transfection. We further monitored EGFP expression driven by PGK promoter over a 5-week period. The results demonstrated that transgene integrated on the three sites could support reliable and stable EGFP expression in IBRS-2 cells, and exhibited a similar expression trend that increased first and then stabilized (30 d) (Fig. 3d). However, it was obvious that EGFP expression on Pifs501, Pifs302 and *pRosa26* was higher overall compared with those on site 13.

### Targeted integrations on Pifs501 site do not induce significant upregulation of nearby genes

**Figure 4:**
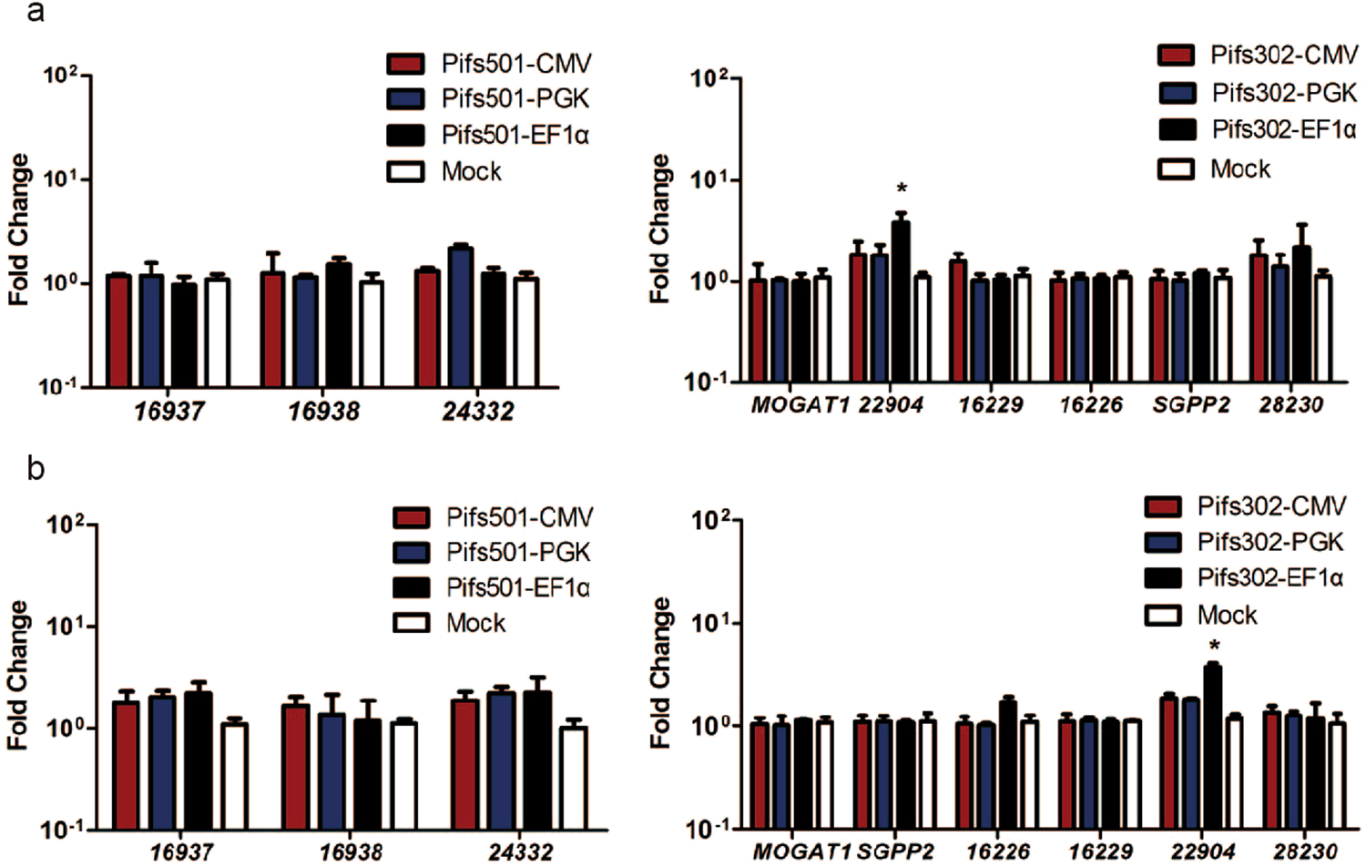
Expression fold change of nearby genes before and after exogenous DNA integration. (**a**) Fold changes in neighboring gene expression of the 600 kb genomic region surrounding Pifs302 and Pifs501 measured by QPCR in IBRS-2 cells. For all genes, n = 3, mean ± SD, and *p < 0.05. Expression of each gene before integration is used as a control. (**b**) Similar analysis to that in a (**a**) is performed in pig fibroblast cells (mean ± SD, n = 3, per promoter per locus). Transfection reagents were only transfected in mock.

Interactions between exogenous DNA and the host genome limit the reliability and safety of transgene integration. The integration of an expression cassette has the possibility of disturbing the expression of adjacent genes^40^. We assessed the impact of EGFP cassette integration on the expression of environment genes 600 kb concentrating on inserted sites by Q-PCR analysis in IBRS-2 cell. For Pifs302 locus, EF1α promoter cassette had significantly (p = 0.03) upregulated gene ENSSSCG00000022904 (Fig. 4a). In contrast to the results above, analysis performed on Pifs501 revealed almost no impact on the expression of flanking genes when we used constitutive expressed CMV, PGK or EF1α promoter (Fig. 4a), the same as the results of *pRosa26* (data not shown). We obtained similar results in pig fetal fibroblast cell (Fig. 4b). Although Pifs302 could support considerable transgene expression, our results revealed that transgene integrated on it did interfere with the expression of nearby genes. From this point of view, Pifs501 site was relatively friendly for exogenous integration.

### Promoters integrated on Pifs501 showed hypomeythlated modification

**Figure 5:**
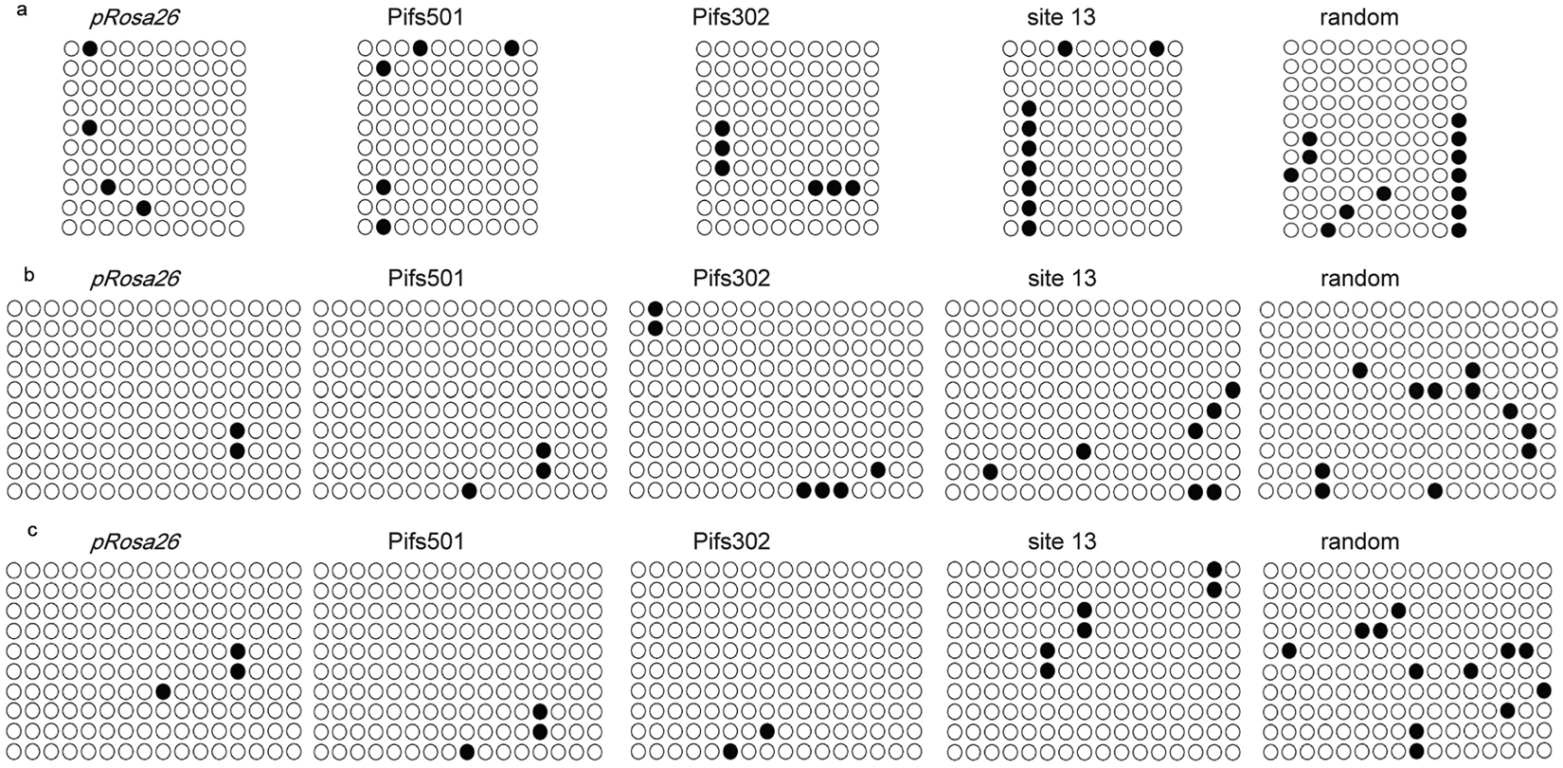
Methylation status of three promoters on different integration loci. (**a**) DNA methylation status in the CMV promoter region on different integration loci detected by bisulfite sequencing. (**b**) DNA methylation status in the PGK promoter region on different integration loci detected by bisulfite sequencing. (**c**) DNA methylation status in the EF1α promoter region on different integration loci detected by bisulfite sequencing. Circles represent CpG sites. Black circles represent that CpG sites are methylated, while blanket circles represent unmethylated CpG sites.

DNA methylation, especially promoter region, regulates genome functions through affecting gene transcription and chromatin formation^41^. Since hypermethylated promoter is prone to cause exogenous gene silencing, the methylation status may constitute a criterion to define optimal friendly harbor loci. Nest PCR was adopted to amplify promoter sequence containing CpG sites from bisulfite converted targeted cell genome. Bisulfite sequencing was then utilized to detect the DNA methylation status of three promoters. We found that DNA methylation status of CMV promoter was higher than that of PGK and EF1α promoters, regardless of site type (Fig. 5a–c). Moreover, Pifs501 and *pRosa26* exhibited lower DNA methylation level of three promoters relative to site 13, while the DNA methylation status of random integration (exogenous EGFP cassette without homologous arms) was the highest, as shown in Fig. 5a–c. These methylation modification patterns of Pifs501 and *pRosa26* were shown to be linked to permissive chromatin for transgene expression.

**Figure 6:**
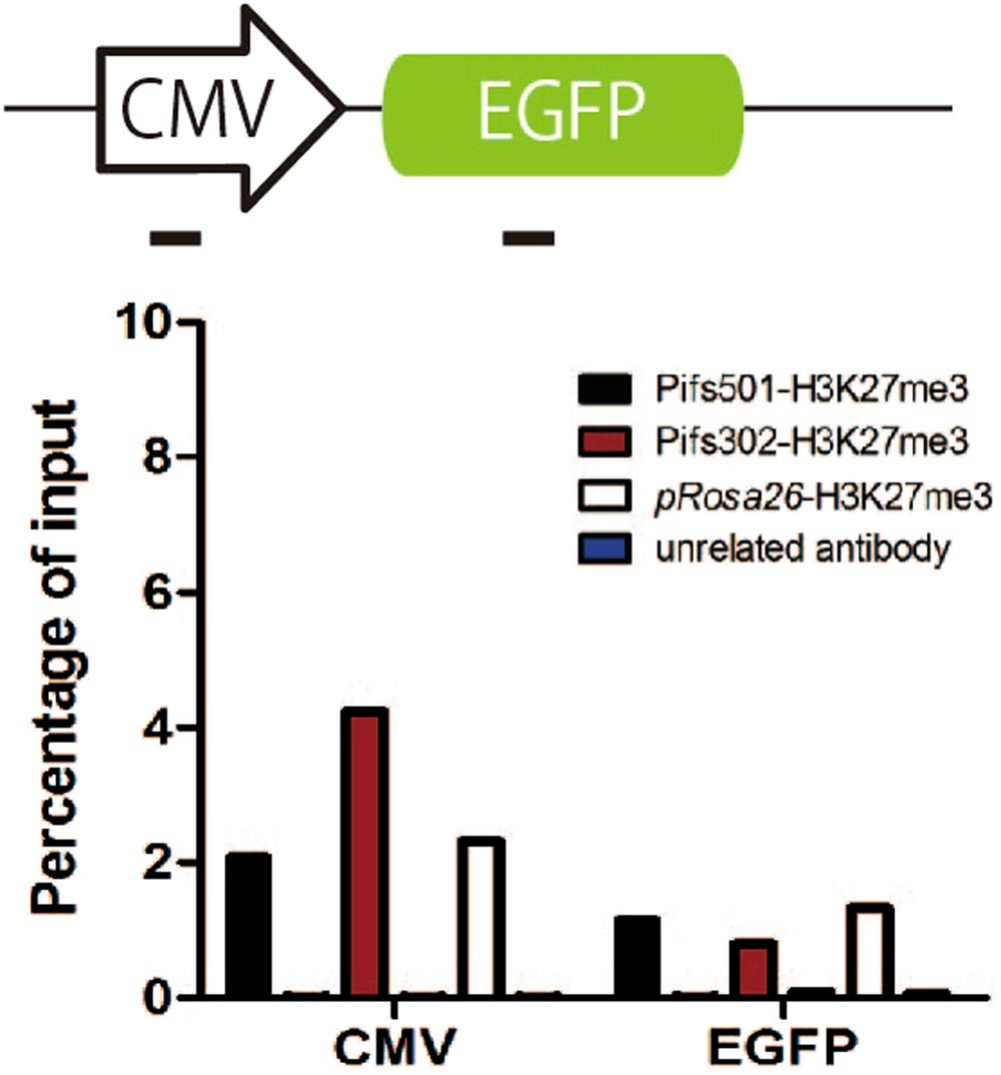
ChIP analysis of H3K27me3 modifications in CMV promoter driven EGFP expression cassettes. Irrelevant antibody used is antibody for human lactoferrin. Shown are means of Q-PCR results from two independent ChIP experiments.

To investigate histone modification of exogenous expression cassettes, we performed chromatin immunoprecipitation (ChIP) analysis on different GFP cassettes in integrated IBRS-2 cells. Histone modification on promoter region and EGFP gene body associated with repressive chromatin (H3K27me3) were mapped. Overall, Pifs501 and *pRosa26* showed less H3K27me3 modification on CMV promoter region (Fig. 6) relative to Pifs302. However, H3K27me3 modification status of other two promoters and EGFP gene bodies among sites was similar (data not shown).

### EGFP expression in transgenic pig and establishment of a versatile cell line for efficient site-specific integration

**Figure 7:**
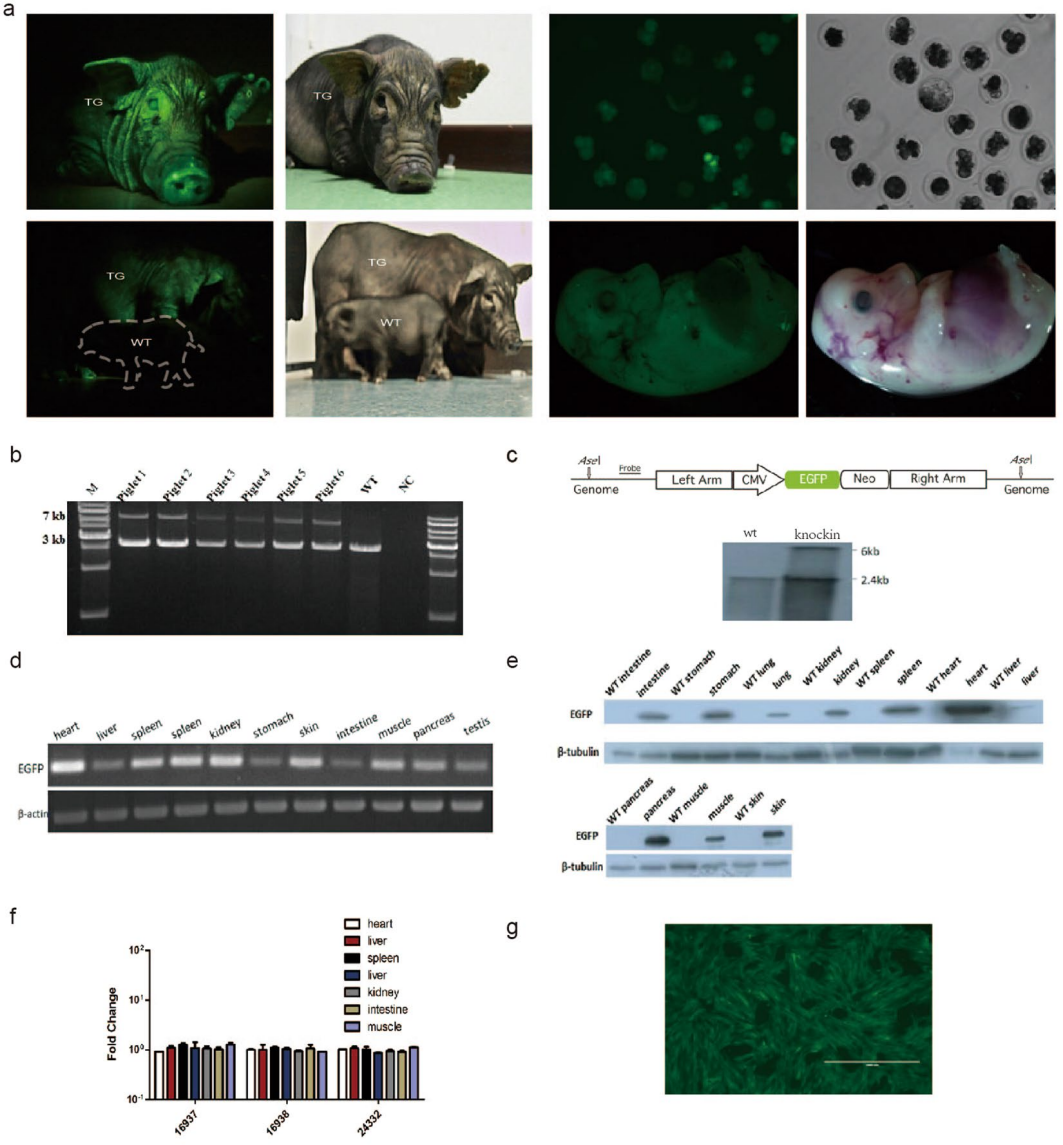
Characterization of EGFP expression in transgenic pigs. (**a**) Pifs501 site-specific integrated transgenic piglets born from SCNT, which were in a healthy state. (**b**) PCR analysis confirmed the correct homologous recombination at the Pifs501 locus in six transgenic piglets. Primer locations are shown in Fig. 2a. (c) Southern blots show targeted integration of EGFP cassettes into Pifs501, with the restriction sites and probes used for analysis indicated as shown. (**d**) RT-PCR analysis of EGFP expression in diverse tissues of transgenic pigs. (**e**) Western blotting analysis of EGFP expression in diverse tissues of transgenic pigs. (**f**) Fold changes in neighboring gene expression of the 600 kb genomic region surrounding Pifs501 measured by QPCR in multiple tissues. For all genes, n = 3, mean ± SD, and p < 0.05. Expression of each gene prior to integration is used as a control. (**g**) Master fibroblasts established from Pifs501 site-specific integrated fetals. The scale bar is 1000 μm.

Pifs501-CMV-EGFP site-specific targeted cells were used for somatic cell nuclear transfer (SCNT). Transgenic piglets were born alive and healthy (Fig. 7a). We found that Pifs501 could support stable expression of exogenous gene EGFP at different development stages, in cloned blastocysts, embryos, and after birth (Fig. 7a). Identification of site-specific integration on Pifs501 was achieved by PCR (Fig. 7b) and southern blotting detection (Fig. 7c, full-length blots are presented in Supplementary Fig. S3). EGFP was ubiquitously expressed in various tissues of site-specific transgenic pig with the highest expression in heart and pancreas (Fig. 7d,e and Fig. S2, full-length blots are presented in Supplementary Fig. S4–9). The relative high EGFP expression in heart and pancreas might be conducive to researches in these two tissues. Notably, EGFP integrated on Pifs501 did not interfere with the expression of neighboring genes within 600 kb in multiple tissues, such as heart, liver, spleen, lung, kidney, muscle and intestine (Fig. 7f). The pregnancy of two pregnant surrogates was terminated at day 35 after embryo transfer to isolate pig fetal fibroblast cells. A high level of EGFP expression was observed in these cells (Fig. 7g). As heterotypic *lox*P flanking EGFP was introduced into these Pifs501-CMV-EGFP master cells, we could replace EGFP cassette with any gene of interest by recombinase-mediated cassette exchange without drug selection. To test the feasibility of this approach and to study whether Pifs501 could support tissue specific gene expression, we engineered an exchange vector containing *Follistatin* regulated by pig muscle specific regulatory elements. This exchange vector, together with a Cre expression plasmid, was transfected into Pifs501-CMV-EGFP master cells, and we obtained single cell clones with EGFP substituted by *Follisatin*. This indicates that other genes can conveniently replace EGFP through Cre/*lox*P system in Pifs501 versatile master cell line. All of the results above indicated that the Pifs501 locus might function as a potential site for exogenous gene expression.

## Discussion

Efficient transgene expression in recipient cells constitutes the primary step in animal model construction and ensures the emergence of relevant phenotypes. Exogenous DNA inserted on a specific genomic site could overcome the significant deficiencies of random integration^42,43^. The current study presented a strategy combining bioinformatics and functional verification to screen transgene integration sites in pig genome. This is of particular importance, given the lack of pluripotent stem cells with considerable proliferation potential in pigs^44^. Using gene expression data of diverse tissues/cell lines, we found actively transcribed regions in pig genome. We excluded areas containing cancer-related genes, as the most common insertional oncogenesis events were due to the transactivation of neighboring tumor-promoting genes^45,46^. To reduce interference to transcriptional units, we focused on intergenic regions with low nucleosome potential. This was based on the fact that nucleosomes play an important role in providing access for regulatory transcription factors to gene regulatory regions, which is essential for activation of gene expression^37^. Finally, we screened out two candidate sites that largely reflect our original intention of friendly integration, and named them Pifs302 and Pifs501.

We performed function verification for candidate sites on the cellular and individual level. It was confirmed that the candidate sites screened by our study could allow site-specific integration of different genetic elements. We obtained sustainable transgene expression from intergenic insertion, eliminating concerns about the feasibility of transgene expression from an intergenic region. EGFP expression was achieved on all loci in IBRS-2 cells and pig fetal fibroblast cells, with Pifs501 and *pRosa26* sites outperforming Pifs302 and site 13. This was probably because Pifs501 and *pRosa26* sites located in a more permissive chromatin environment. Pifs501 site-specific integrated transgenic pigs were healthy, and EGFP was ubiquitously expressed in diverse tissues. EGFP expression in other tissues is lower than that in heart and pancreas, probably due to the different DNA methylation status of CMV promoter in different tissues. In addition, Pifs501 supported EGFP expression in different development stages, such as the blastocyst stage, the embryonic stage, and the after-birth stage. Furthermore, transgenic pigs were fertile, and they successfully produced the next generation. This indicated that there was no evident potential genotoxic effect at the level of the organism. The fetal fibroblast master cell line that we established contained two *lox P* sequences on the Pifs501 flanking EGFP, which could be utilized to replace EGFP to any other gene of interest through a recombinase mediated cassette exchange reaction with Cre/*lox*P system. As in the case of the substitution we performed, we successfully exchanged EGFP to *Follistatin* using RMCE technology.

We provided an initial, but important safety test for candidate sites. Our data did not reveal any significantly dysregulated gene expression 600 kb surrounding Pifs501 when we used constitutively expressed CMV, PGK and EF1α promoter. This suggested that integration on Pifs501 might have no detrimental impact on the expression of nearby genes. However, integration on Pifs302 dysregulated the expression of one neighbouring gene, probably because Pifs302 located in a relatively gene-dense area. Chromatin segments with active markers are more likely to be safe sites, as they are predictive of high transcriptional activity and suitable for transgene expression. Compared with other loci, hypomethylated status of promoter on Pifs501 and *pRosa26* might endow higher EGFP expression on them in IBRS-2 cells. Epigenetic modification investigation demonstrated that Pifs501 and *pRosa26* might locate in a relative open chromosomal area, which was convenient for better access of transcriptional elements.

With limited knowledge of the reciprocal interaction between exogenous integrated DNA and the host genomic context, researchers have not yet reached a consensus regarding defining a genomic safe harbor. Moreover, genomes are spatially organized at multiple scales, from packaging of DNA around individual nucleosomes to segregation of whole chromosomes into distinct territories^47–49^. With more information emerging on three-dimensional genome’s dynamic packaging organization and genomic function, determining precisely how longer-range interactions work is becoming increasingly clear^50^. However, final determination of the genomic safe harbor for effective transgene integration should be subject to long-term functional studies with different genetic elements and in more cell types. The good performance of transgene expression on Pifs501 in our experiment indicated that the integration sites screen strategy that we proposed was effective. Through our *in vitro* and *in vivo* functional assays, we could conclude Pifs501 as a potential genomic docking site for transgene insertion in pigs. Certainly, efficient expression of other genes on this site, and the expression in the transgenic animal offspring needs further investigation.

## Method

All experiments were performed in accordance with relevant guidelines and regulations. All experiments were approved by the Animal Care and the Use Committees of the State Key Laboratories for Agrobiotechnology, College of Biological Sciences, China Agricultural University.

### Vector construction

We constructed a homologous recombination targeting vector for each candidate site. For Pifs501, the primer sequences for the 5′ arm were 5′ AAGTGGAGCCAAACCAATCCAT3′ (forward) and 5′GCTACAACTTCCCTGTCAATC3′ (reverse), and 5′CTCAGAAGTGTATTGACAG3′ (forward) and 5′ GAGCTTTCAACCATTCTGGCC 3′ (reverse) for the 3′ arm. For Pifs302, the primer sequences for the 5′ arm were 5′GGACGCCTCACAATCATCCTAT3′ (forward) and 5′ GTGTGTCGCCAATTCTATAGAA 3′ (reverse), and 5′CAAGTGCTGAGCTCCCAGGCAG 3′ (forward) and 5′TCAAAGTGTGGCCATGGGTGGCC3′ (reverse) for the 3′ arm. The left and right homologous arms for Pifs501 are 899 bp and 1873 bp respectively, while the left and right homologous arms for Pifs302 are 922 bp and 2009 bp respectively.

### Cell culture and single cell clone screen

IBRS-2 cells and pig fetal fibroblast cells were cultured in DMEM (Gibco, 11960) +10% FBS (Gibco, 10099). 1 × 10^6^ cells were transfected with EGFP expressing targeting vector and the corresponding Cas9 vector (a total of 4 µg) using lonza electroporation reagent (VPI-1002) by lonza electroporation procedure A024. Targeting plasmid was linearized using SalI restriction enzyme (NEB). After transfection, the cells were plated on 20 10-cm dishes and selected by 800 µg/ml G418 (CALBIOCHEM, 345810) for 8~12 d. Single cell colonies were picked using cloning cylinders, cultured in 48-well plates, and screened by PCR analysis.

### Identification of positive site-specific integrations

Genomic DNA of cell clones was extracted with the Blood and Cell Culture DNA Midi kit (Qiagen, 69506). For gene targeting analysis, genomic DNA of single cell clones was analyzed by PCR detection. Primer sequences spanning the whole targeting region are as follows: for Pifs501, 5′ TGTGGACTGTTGGCA AAG 3′ (forward) and 5′ AAGACCTGGAAGAACTGGC3′ (reverse). For Pifs302, 5′ CCTGATCCTTGGGGCAGAGC 3′ (forward) and 5′ TTGGGTTTAGCAGCCCTT’ (reverse). For southern blot analyses, 10 µg genomic DNA of transgenic pig was digested with AseI (NEB). The hybridization probe used to detect the GFP transcription unit DNA (GFP probe) was synthesized by PCR, and the sequence of the primer was 5′ TGTGGACTGTTGGCAAAG3′ (forward) and 5′AGGTATTAGGGTGGGTATTCAC’ (reverse).

### Gene expression analysis

Total RNAs extracted from each sample using the RNeasy Mini kit (Qiagen, 74106) were reverse-transcribed to cDNA according to the manufacturer’s instructions (Promega, M1705). Real-time PCR was performed using SYBR Premix Ex Taq (TaKaRa, RR820A) and the 7500 Real-Time PCR System (Applied systems), with the following parameters: 95 °C for 30 s, followed by 40 two-step cycles at 95 °C for 5 s and at 60 °C for 4 s. Primers for EGFP were 5′CAGAAGAACGGCATCAAGGT3′(forward) and 5′TGGGTGCTCAGGTAGTGGTT 3′(reverse). β-actin was used as a reference gene, and primer sequences for β-actin were 5′TGGACATCAGGAAGGACCTC3′ (forward) and 5′ACATCTGCTGGAAGGTGGAC3′ (reverse). Primers for GAPDH were 5′GTCGGTTGTGGATCTGACCT3′ (forward) and 5′ GTCCTCAGTGTAGCCCAGGA3′ (forward). The relative expression level of each gene was calculated by the ΔΔCt method, normalized to β-actin and GAPDH expression (housekeeping gene controls).

### Bisulfite sequencing

Bisulfite convertion was performed on 0.5 µg of genomic DNA from site-specific integrated cell clones using the MethylDetector Kit (ACTIVE MOTIF, 55001), according to the manufacturer’s instructions. Bisulfited-modified DNA was used to amplify the promoter fragments by nested PCR using Taq DNA polymerase (Kangwei, CW0680F), with the following conditions: 94 °C for 5 min, followed by 35 three-step cycles at 94 °C for 30 s, 52 °C for 30 s, and 72 °C for 20 s. The PCR products of the first round were used as a template for second round amplification with the same condition. The final PCR products were separated on 2% agarose gels and purified, followed by TA cloning and sequencing. The presence of a cytosine residue after bisulfite treatment shows that the cytosine residue was protected from bisulfite modification by methylation. The primers were listed in Table S5.

### Chromatin immunoprecipitation analysis

Site-specific integrated IBRS-2 cells cultured in 10 cm plates were cross linked with 1% formaldehyde, and analyzed using a ChIP assay kit (P2078, Beyotime) according to the manufacturer’s instructions. Genome DNA was fragmented by sonication. DNA-protein complexes were immunoprecipitated with the following antibodies: anti-H3K27me3 (abcam, ab6002). Unrelated antibody used is antibody for human lactoferrin (Sigma, L4894). After removing the protein, DNA was purified and qPCR was performed.

### Statistical analysis

We employed the GraphPad Prism 5 software to conduct statistical analysis. Results are expressed as the mean ± SD. Unless otherwise indicated, differences between experimental groups were compared using an unpaired two-tailed Student’s t-test. P value < 0.05 was considered statistically significant.

## Electronic supplementary material

supplementary information

## References

[1] Cartier N (2009). Hematopoietic Stem Cell Gene Therapy with a Lentiviral Vector in X-Linked Adrenoleukodystrophy. Science.

[2] Biffi A (2013). Lentiviral Hematopoietic Stem Cell Gene Therapy Benefits Metachromatic Leukodystrophy. Science.

[3] Schroder ARW (2002). HIV-1 integration in the human genome favors active genes and local hotspots. Cell.

[4] Yang DS (2010). Expression of Huntington’s disease protein results in apoptotic neurons in the brains of cloned transgenic pigs. Hum Mol Genet.

[5] Garrick D, Fiering S, Martin DIK, Whitelaw E (1998). Repeat-induced gene silencing in mammals. Nat Genet.

[6] Giraldo P, Rival-Gervier S, Houdebine LM, Montoliu L (2003). The potential benefits of insulators on heterologous constructs in transgenic animals. Transgenic Res.

[7] McBurney MW, Mai T, Yang XF, Jardine K (2002). Evidence for repeat-induced gene silencing in cultured mammalian cells: Inactivation of tandem repeats of transfected genes. Exp Cell Res.

[8] Carter DB (2002). Phenotyping of transgenic cloned piglets. Cloning and stem cells.

[9] Rogers CS (2008). Disruption of the CFTR gene produces a model of cystic fibrosis in newborn pigs. Science.

[10] Aigner B (2010). Transgenic pigs as models for translational biomedical research. Journal of molecular medicine.

[11] Chang AH, Sadelain M (2007). The genetic engineering of hematopoietic stem cells: The rise of lentiviral vectors, the conundrum of the LTR, and the promise of lineage-restricted vectors. Mol Ther.

[12] Brinster RL (1989). Targeted Correction Of a Major Histocompatibility Class-Ii E-Alpha-Gene by DNA Microinjected into Mouse Eggs. P Natl Acad Sci USA.

[13] Donoho G, Jasin M, Berg P (1998). Analysis of gene targeting and intrachromosomal homologous recombination stimulated by genomic double-strand breaks in mouse embryonic stem cells. Mol Cell Biol.

[14] Urnov FD, Rebar EJ, Holmes MC, Zhang HS, Gregory PD (2010). Genome editing with engineered zinc finger nucleases. Nat Rev Genet.

[15] Christian M (2010). Targeting DNA Double-Strand Breaks with TAL Effector Nucleases. Genetics.

[16] Miller JC (2011). A TALE nuclease architecture for efficient genome editing. Nat Biotechnol.

[17] Shao YJ (2014). CRISPR/Cas-mediated genome editing in the rat via direct injection of one-cell embryos. Nat Protoc.

[18] Sadelain M, Papapetrou EP, Bushman FD (2012). Safe harbours for the integration of new DNA in the human genome. Nat Rev Cancer.

[19] Papapetrou EP (2011). Genomic safe harbors permit high beta-globin transgene expression in thalassemia induced pluripotent stem cells. Nat Biotechnol.

[20] Irion S (2007). Identification and targeting of the ROSA26 locus in human embryonic stem cells. Nat Biotechnol.

[21] Li XP (2014). Rosa26-targeted swine models for stable gene over-expression and Cre-mediated lineage tracing. Cell Res.

[22] Yang, D. S. *et al*. Identification and characterization of rabbit ROSA26 for gene knock-in and stable reporter gene expression. *Sci Rep-Uk* **6**, doi:Artn 2516110.1038/Srep25161 (2016). 10.1038/srep25161 PMC4846827 27117226

[23] Wu, M. M. *et al*. Rosa26-targeted sheep gene knock-in via CRISPR-Cas9 system. *Sci Rep-Uk* **6**, doi:Artn 2436010.1038/Srep24360 (2016). 10.1038/srep24360 PMC4827023 27063570

[24] Kong Q (2014). Rosa26 locus supports tissue-specific promoter driving transgene expression specifically in pig. PloS one.

[25] Zambrowicz BP (1997). Disruption of overlapping transcripts in the ROSA beta geo 26 gene trap strain leads to widespread expression of beta-galactosidase in mouse embryos and hematopoietic cells. Proceedings of the National Academy of Sciences of the United States of America.

[26] Versteeg R (2003). The human transcriptome map reveals extremes in gene density, intron length, GC content, and repeat pattern for domains of highly and weakly expressed genes. Genome Res.

[27] Hippenmeyer S (2010). Genetic Mosaic Dissection of Lis1 and Ndel1 in Neuronal Migration. Neuron.

[28] Ruan J (2015). Highly efficient CRISPR/Cas9-mediated transgene knockin at the H11 locus in pigs. Sci Rep.

[29] Freeman, T. C. *et al*. A gene expression atlas of the domestic pig. *Bmc Biol* **10**, doi:Artn 90. 10.1186/1741-7007-10-90 (2012). 10.1186/1741-7007-10-90 PMC3814290 23153189

[30] Riviere I, Dunbar CE, Sadelain M (2012). Hematopoietic stem cell engineering at a crossroads. Blood.

[31] Jacquier A (2009). The complex eukaryotic transcriptome: unexpected pervasive transcription and novel small RNAs. Nat Rev Genet.

[32] Burge SW (2013). Rfam 11.0: 10 years of RNA families. Nucleic Acids Res.

[33] Lagesen K (2007). RNAmmer: consistent and rapid annotation of ribosomal RNA genes. Nucleic Acids Res.

[34] Lestrade L, Weber M (2006). J. snoRNA-LBME-db, a comprehensive database of human H/ACA and C/D box snoRNAs. Nucleic Acids Res.

[35] Griffiths-Jones S (2006). miRBase: the microRNA sequence database. Methods in molecular biology.

[36] Chan PP, Lowe TM (2009). GtRNAdb: a database of transfer RNA genes detected in genomic sequence. Nucleic Acids Res.

[37] Henikoff S (2009). Labile H3.3 + H2A.Z nucleosomes mark ‘nucleosome-free regions’. Nat Genet.

[38] Jin CY (2009). H3.3/H2A.Z double variant-containing nucleosomes mark ‘nucleosome-free regions’ of active promoters and other regulatory regions. Nat Genet.

[39] Levitsky VG (2004). RECON: a program for prediction of nucleosome formation potential. Nucleic Acids Res.

[40] Lombardo A (2011). Site-specific integration and tailoring of cassette design for sustainable gene transfer. Nat Methods.

[41] Bird AP, Wolffe AP (1999). Methylation-induced repression - Belts, braces, and chromatin. Cell.

[42] Bronson SK (1996). Single-copy transgenic mice with chosen-site integration. P Natl Acad Sci USA.

[43] Costantini F, Lacy E (1981). Introduction Of a Rabbit Beta-Globin Gene into the Mouse Germ Line. Nature.

[44] Brevini TAL, Antonini S, Cillo F, Crestan M, Gandolfi E (2007). Porcine embryonic stem cells: Facts, challenges and hopes. Theriogenology.

[45] Hacein-Bey-Abina S (2003). LMO2-associated clonal T cell proliferation in two patients after gene therapy for SCID-X1. Science.

[46] Kustikova O (2005). Clonal dominance of hematopoietic stem cells triggered by retroviral gene marking. Science.

[47] Bickmore WA (2013). The Spatial Organization of the HumanGenome. Annu Rev Genom Hum G.

[48] Gibcus JH, Dekker J (2013). The Hierarchy of the 3D Genome. Mol Cell.

[49] Levine M, Cattoglio C, Tjian R (2014). Looping Back to Leap Forward: Transcription Enters a New Era. Cell.

[50] Boettiger AN (2016). Super-resolution imaging reveals distinct chromatin folding for different epigenetic states. Nature.

