# Supplementary material for: Screen and Verification for Transgene Integration Sites in Pigs
Source: Sci Rep. 2018 May 9;8:7433. doi: 10.1038/s41598-018-24481-1 (PMC5943519; doi:10.1038/s41598-018-24481-1)
Supplement: Supplementary file 1 — supplementary information [file 41598_2018_24481_MOESM1_ESM.pdf]

Full Title:
Screen and Verification for Transgene Integration Sites in Pigs

Author information:
Linyuan Ma[1], Yuzhe Wang[1], Haitao Wang[1], Yiqing Hu[1], Jingyao Chen[1], Tan Tan[1], Man Hu[1], Xiaojuan Liu[1], Ran Zhang[1], Yiming Xing[1], Yiqiang Zhao[1,2]*, Xiaoxiang Hu[1], Ning Li[1]*.

Table S1. Candidate intergenic regions without noncoding RNAs.

Chr.	Region
chr_18	33510000-33515000
chr_18	33660000-33665000
chr_18	33905000-33910000
chr_18	33930000-33935000
chr_16	44285000-44290000
chr_15	137955000-137960000
chr_15	138345000-138350000

Table S2. All intergenic regions between candidate 500 kb windows.

Chr.	Region
chr_18	33510000-33515000
chr_18	33660000-33665000
chr_18	33905000-33910000
chr_18	33930000-33935000
chr_15	137955000-137960000
chr_15	138050000-138055000
chr_15	138064000-138069000
chr_15	138345000-138350000
chr_15	138462000-138467000
chr_16	44190000-44195000
chr_16	44285000-44290000

(5kb intergenic regions adjacent to genes were considered as candidates.)

Table S3. sgRNAs targeting multiple sites in 1 kb candidate intergenic regions.

Chr.	Sequence
chr_15	GTTGCTGCGG AGACAGTGAC
chr_15	GACACACAAGTGCTGAGCTCCC
chr_15	GTAGACAAGGGATGCTAGGA
chr_15	GTTAGTAATA ATGGATATAG
chr_15	GAGCATTCTGTGTCTTCTGCA
chr_16	GTTGTAGCTCATAACTTCTCG
chr_16	GAGTTCCGTTTAGTTAAGGA
chr_16	GCCAAAGGTAATGCATGGTA
chr_16	GTCAAAGAATTGCAGAGCAAGA
chr_18	GTCTTAGGCCACCCCTCCTGA
chr_18	GTCTTTGGGT CATGATCAAT GAA

Table S4. Potential off-target cleavage sites of Pifs501.

Site	Chr.	Range
1	chr_16	23387960-23387946
2	chr_16	12420941-12420929
3	chr_16	21785005-21794993

Table S5. PCR primers used in nested PCR in the amplification of promoter fragments.

Primer	Sequence
PCR amplification for CMV promoter (first round)	me-cmv-F1: 5’AATTGTTTATTTGGTAGTATATTAAGTGTA3’,
	me-cmv-R1: 5’AATTGTTTATTTGGTAGTATATTAAGTGTA3’
PCR amplification for CMV promoter (second round)	me-cmv-F2: 5’AGTATATTAAGTGTATTATATGTTAAGTA3’,
	me-cmv-R2: 5’ ATAAAATAAAAACTTAAAAATCCCC3’;
PCR amplification for PGK promoter (first round)	me-pgk-F1: 5’ TTTTTAAGGTAGTTTGGAGTAT
	me- pgk -R1: 5’ TTCCCAACCT CTAAACCCAA A
PCR amplification for PGK promoter (second round)	me- pgk -F2: 5’ TTATATAAGTGGTTTTTGGTTT3’,
	me- pgk -R2: 5’ AAAACCAAAC TACTATTAAC3’;
PCR amplification for ef1α promoter (first round)	me-ef1α-F1: 5’ GTTTGTTTTGGTGTTTGGTTT3’
	me- ef1α-R1: 5’ ACTTTAAACC ACTATCTAAA ACT3’
PCR amplification for ef1α promoter (second round)	me- ef1α-F2: 5’ GGTGAGTTATTTATATAAAGGAA3’
	me- ef1α-R2: 5’ ACATCAAATA CCAAACTAAC C3’

primers were designed by an online website:
(http://www.urogene.org/cgi-bin/methprimer/methprimer.cgi).

Table S6. Numbers of single cell clones

Cell type	Site	Promoter	Number of single cell clones
IBRS-2	Pifs501	CMV	83
IBRS-2	Pifs501	PGK	69
IBRS-2	Pifs501	EF1α	78
IBRS-2	Pifs302	CMV	76
IBRS-2	Pifs302	PGK	83
IBRS-2	Pifs302	EF1α	56
IBRS-2	*pRosa26*	CMV	50
IBRS-2	*pRosa26*	PGK	43
IBRS-2	*pRosa26*	EF1α	73
IBRS-2	Site 13	CMV	50
IBRS-2	Site 13	PGK	52
IBRS-2	Site 13	EF1α	58
Pig fibroblast	Pifs501	CMV	62
Pig fibroblast	Pifs501	PGK	76
Pig fibroblast	Pifs501	EF1α	132
Pig fibroblast	Pifs302	CMV	90
Pig fibroblast	Pifs302	PGK	89
Pig fibroblast	Pifs302	EF1α	48
Pig fibroblast	*pRosa26*	CMV	68
Pig fibroblast	*pRosa26*	PGK	67
Pig fibroblast	*pRosa26*	EF1α	73
Pig fibroblast	Site 13	CMV	47
Pig fibroblast	Site 13	PGK	56
Pig fibroblast	Site 13	EF1α	61

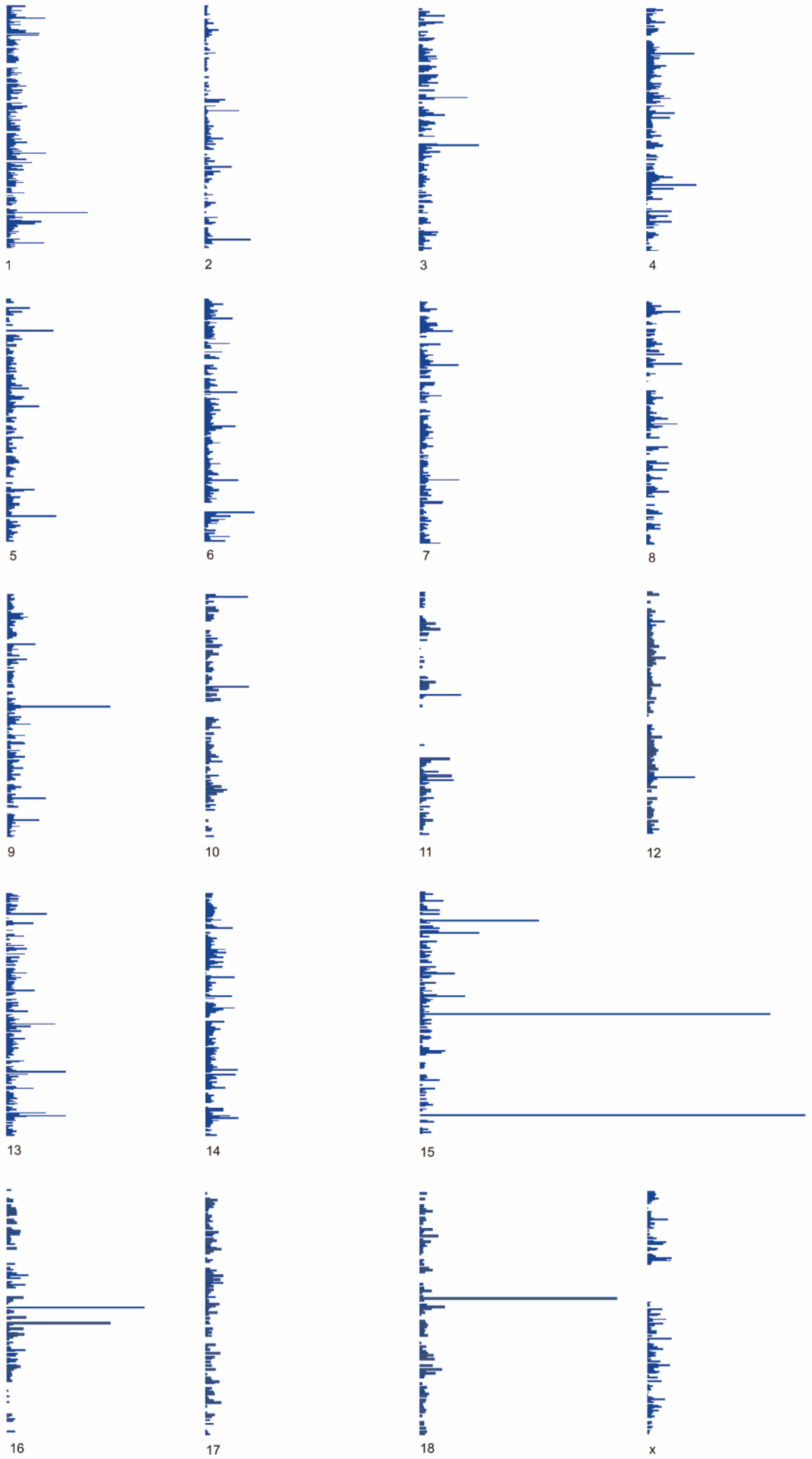

Figure S1. Average gene expression profiles of 500 kb windows for pig chromosomes.

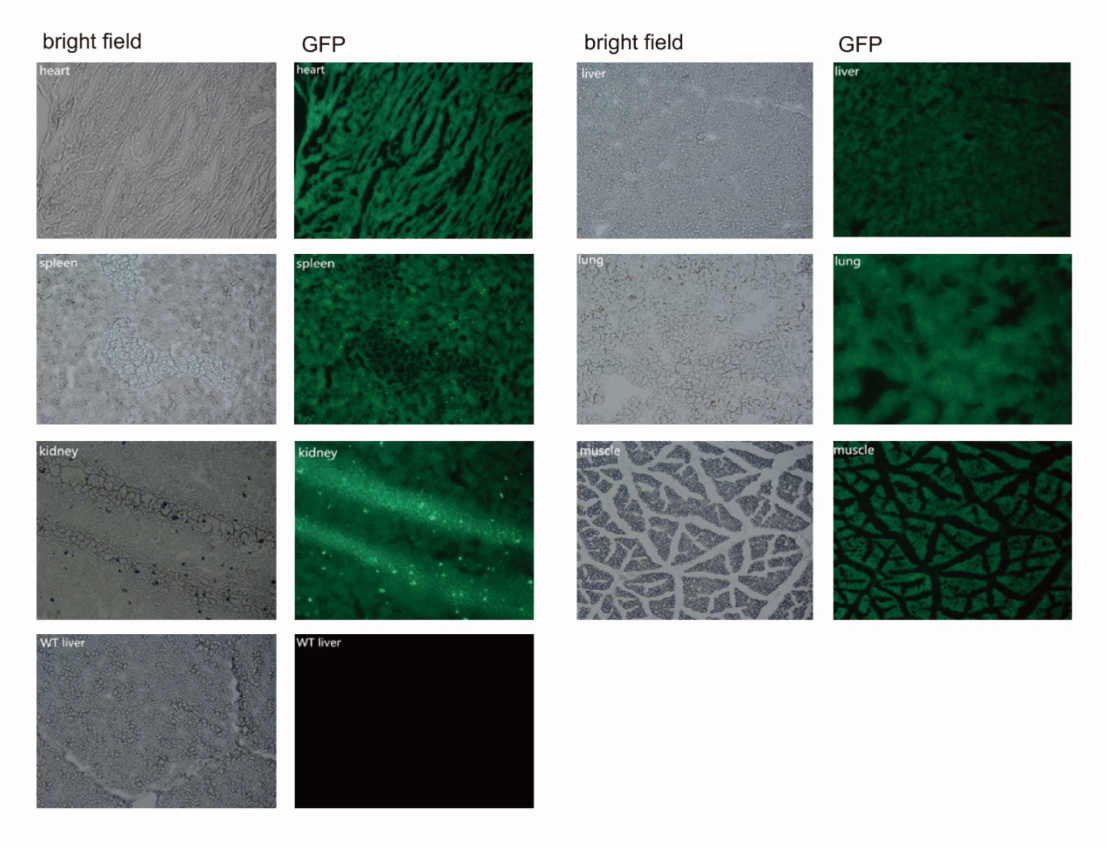

Figure S2. EGFP expression analysis of frozen tissue sections in diverse tissues of transgenic pigs.

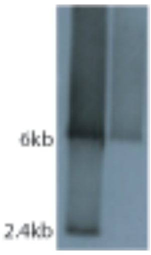

Figure S3. Full length blot responding to Fig. 7C.

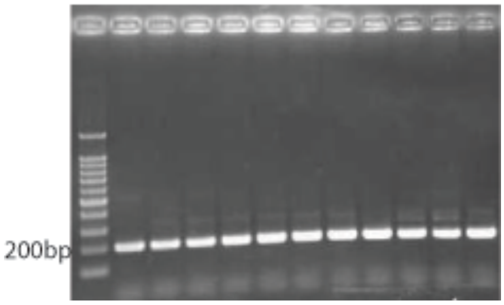

Figure S4. Full length blot responding to Fig. 7D.

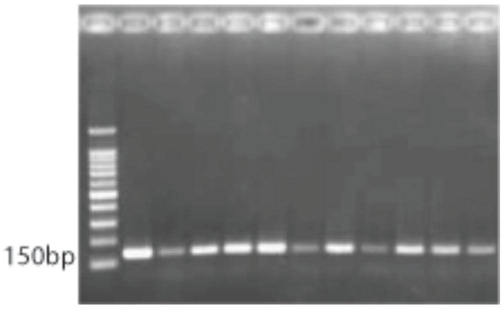

Figure S5. Full length blot responding to Fig. 7D. The up EGFP lane.

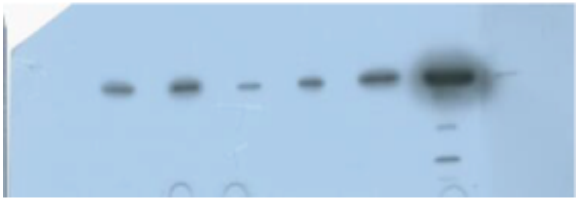

Figure S6. Full length blot responding to Fig. 7E.

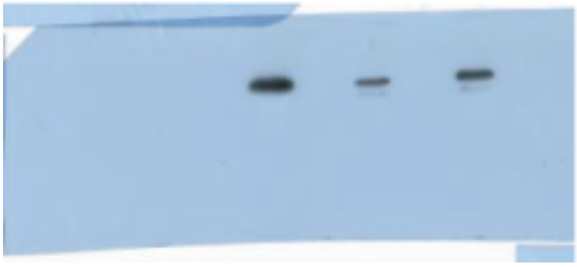

Figure S7. Full length blot responding to Fig. 7E.

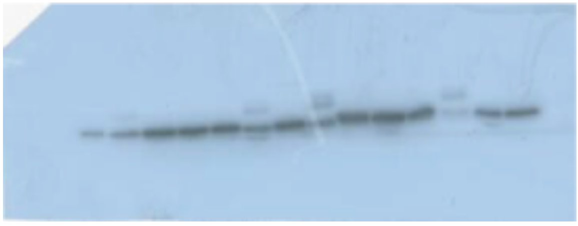

Figure S8. Full length blot responding to Fig. 7E.

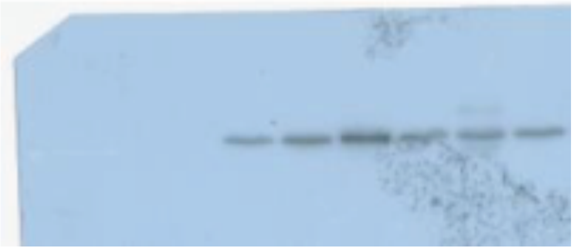

Figure S9. Full length blot responding to Fig. 7E.
